# Ebola Virus RNA Persists in Reproductive Tissues and Sperm of Male *Ifnar1*^−/−^ Mice After Resolution of Viremia

**DOI:** 10.3390/v18090983

**Published:** 2026-09-07

**Authors:** Andrew L. Webb, Brayden G. Schindell, Kathy Frost, Geoff Soule, Stephanie A. Booth, David Safronetz, Jason Kindrachuk

**Affiliations:** 1 Department of Medical Microbiology and Infectious Diseases, University of Manitoba, Winnipeg, MB R3T 2N2, Canada; webba2@myumanitoba.ca (A.L.W.);; 2 Mycobacteriology, Vector-Borne and Prion Diseases Division, National Microbiology Laboratory, Public Health Agency of Canada, Winnipeg, MB R3E 3R2, Canada; 3 Special Pathogens Program, National Microbiology Laboratory, Public Health Agency of Canada, Winnipeg, MB R3E 3R2, Canada; 4 Department of Internal Medicine, University of Manitoba, Winnipeg, MB R3A 1R9, Canada; 5 Manitoba Centre for Proteomics and Systems Biology, Health Science Centre, Winnipeg, MB R3A 1R9, Canada

**Keywords:** Ebola virus, *Ifnar1*^−/−^ mice, histopathology, persistent infection, testicular infection

## Abstract

Ebola virus (EBOV) has been shown to persist in the reproductive tracts of male Ebola virus disease (EVD) survivors ranging from months to years following recovery, with shedding of EBOV in semen leading to chains of disease transmission. In the current study, we used a mouse model of EBOV infection to investigate the timing of virus infiltration of the male reproductive tissues, the extent of virus persistence in these tissues, and the impact of persistence on tissue health. We challenged male *Ifnar*^−/−^ mice with wild-type EBOV and collected blood, liver, testis, epididymis, and sperm samples longitudinally for 35 days. All EBOV-challenged mice that were tested 3 days post-infection were viremic, and EBOV RNA was detected in reproductive samples up to 35 days post-infection. Epididymis and sperm samples were notable for detection of EBOV RNA at low cycle thresholds on day 3, and for high frequency of detection in samples up to 21 days post-infection. Testicular tissue histopathology was relatively mild during acute infection, but perturbations of interstitial tissue continued up to 35 days post-infection. Our findings support previous evidence of the risk of sexual transmission of persistent EBOV infections and highlight the potential impact of these infections on male fertility.

## 1. Introduction

*Orthoebolavirus zairense* (Ebola virus; EBOV) is a highly infectious zoonotic pathogen responsible for recurrent Ebola virus disease (EVD) outbreaks in Central and Western Africa, and is associated with high morbidity and mortality in humans [1]. During the acute phase of EVD, early symptoms of illness are nonspecific and can include joint pain, sore throat, abdominal pain, diarrhea, fever, headache, weakness, rash, and sore muscles [2]. In fatal infections, symptoms may escalate and increase in severity, including multi-organ injury (e.g., kidney failure), central nervous system consequences, rapid or shallow breathing, confusion, and bleeding from the nose, mouth, or anus [2,3]. EVD survivors also report a range of ongoing symptoms, such as muscle and joint pain, headache, and fatigue, and these can be musculoskeletal, gastrointestinal, neurological, cardiac, ophthalmological, auditory, or constitutional in nature [4,5,6]. One or more of these symptoms may be caused in part by persistent EBOV infection in specific tissues, including the male reproductive tract, despite its clearance from the circulatory system [7,8].

EBOV persistence in survivors is most commonly determined based on detection of viral material in one or more bodily fluids, which are more readily accessible than tissue samples. EBOV genomic material is most commonly detected in semen, where the virus may persist over a broad time range from months to years after recovery [6,9]. In one study, EBOV RNA was detected in 75.4% of semen samples of disease survivors six months after discharge, and in another study 30% of males tested positive for EBOV genomic material in one or more samples, ranging from 233 to 1178 days (median, 551 days 19 months) [6,9]. However, relatively few details are available regarding the male reproductive tract as a reservoir for persistent EBOV infection. For example, there is a lack of data regarding the timing of EBOV dissemination into testicular tissues, specific cell tropism, and viral replication kinetics during and after acute infection [10,11]. In the solitary case where testis tissue was available from a fatal case of human EVD, the EBOV antigen was present in the seminiferous tubules and interstitial space without histological lesions [12]. The invasive nature of testicular biopsies precludes tissue sampling of male EVD survivors, but animal model experiments of EVD report the presence of viral genomic material in the reproductive tracts of male monkeys, mice, ferrets and guinea pigs during acute and/or persistent infection [13,14,15,16]. Animal models also facilitate investigation into subclinical perturbations of the male reproductive tract during and after EVD. Although the frequency and magnitude of inflammation and/or damage to the structure of the seminiferous tubules and/or epididymis ducts is minimal, there is a lack of data regarding the impact of acute and persistent EBOV infection on signaling factors that maintain the equilibrium between immune tolerance and spermatogenesis [13,14,17,18].

As nonhuman primates most closely parallel humans in terms of EBOV pathogenesis, they are considered the gold standard for studying pathogenesis [10,19]. However, EBOV infection is highly lethal in NHPs, which is an acceptable outcome in studies pertaining to acute EVD but a hindrance in studies of viral persistence in survivors [20,21,22]. While Zeng et al. (2017) describe EBOV persistence in nonhuman primates, they used archived tissues that limited the availability samples collected in a longitudinal series [13]. Mouse models of EVD benefit from greater sample populations, and lethality can be manipulated via mouse or virus modifications [14,23,24]. For example, specific strains of collaborative cross mice are susceptible to mouse-adapted EBOV and resistant to wild-type EBOV, which facilitates identification of markers associated with host mortality [14]. Meanwhile, type 1 interferon receptor knockout mice (*Ifnar1*^−/−^) challenged with wild-type EBOV exhibit symptoms similar to those of human EVD and display variable lethality [23,25]. Previous studies indicate that EBOV disseminates into the male reproductive tract, most commonly in the epididymis, followed by the seminiferous tubules and surrounding tissues that comprise the testis [14,17]. Mouse models have also provided preliminary data confirming the feasibility of intravaginal EBOV infection [14].

In the current study we used a mouse model to investigate EBOV dissemination into—and persistence within—the male reproductive tract, as well as the subsequent impact on reproductive health. We challenged *Ifnar1*^−/−^ mice with EBOV and collected tissue and fluid samples intermittently up to 35 days post-infection. We tested the frequency of detection and change in viral load in blood, liver, testis (i.e., seminiferous tubules and surrounding tissue), epididymis, and sperm samples over time. We also examined the histopathology of liver and testis sections for a subset of mice.

## 2. Materials and Methods

### 2.1. Experimental Design

Experiments were performed using 56 *Ifnar1*^−/−^ mice (male B6.129S2-Ifnar1tm1Agt/Mmjax; The Jackson Laboratory, Bar Harbour, ME, USA) at four to six weeks of age. It was noted that prior to the experiment start point, the mice possessed overgrown teeth that hindered food intake. This issue was resolved by filing down the teeth and the mice returned to regular eating habits. Fifty-three mice were infected with 0.1 pfu of EBOV Makona (H.sapiens-wt/GIN/2014/Makona-Gueckedou-C07, called rgEBOV-C07) by intraperitoneal injection as described previously [23,26]. Serial sampling was performed intermittently up to 35 days post-infection; eight animals were euthanized per time point. Three naïve mice were euthanized on day 14 of the experiment to serve as healthy controls. Blood samples were collected to determine successful infection in challenge mice, and to provide a timeline of acute illness. Liver samples were collected to provide an indicator of disease severity [27]. Sperm and tissue samples were collected from the testis.

### 2.2. Reverse Transcription PCR

At each time point, one of each type of sample (i.e., blood, liver, testis, epididymis, and sperm) was tested for each of eight mice. EBOV RNA was extracted from tissue and fluid samples using the QIAamp Viral RNA Mini Kit (QIAGEN, Hilden, Germany). Detection of EBOV RNA was achieved by RT-PCR using primer pairs specific to EBOV NP [23,28].

### 2.3. Statistical Analysis

Significant differences in time points were determined using the one-way ANOVA test, and significant changes in mean cycle threshold between time points were determined using Student’s *t*-test, for which equality of variance was determined using the F-test. The cutoff for the minimum threshold of detection was 36 cycles. The minimum significance threshold was adjusted using the Bonferroni correction.

### 2.4. Tissue Processing and Imaging

At each time point, one each of liver and testis tissues for each of three mice were processed for histopathological comparison. Tissues were fixed and processed according to Canadian Science Centre for Human and Animal Health guidelines for CL4 pathogens. Samples destined for histological analysis were stained and counterstained with hematoxylin and eosin. Microscopy was performed using the Axio Scan.Z1 slide scanner (Zeiss, Jena, Germany) with a HV-F202SCL front camera (Hitachi, Tokyo, Japan) and an ORCA-Flash4.0 V3 Digital CMOS side camera (Hamamatsu Phototonics, Hamamatsu, Japan). Image analysis was performed using the Fiji package of the software ImageJ (version 1.54p [29,30]). For each experimental sample time point, tissue histopathology was compared against the preceding time point and against naïve mouse tissues collected 14 days post-infection.

## 3. Results

### 3.1. Ebola Virus Infection and Mouse Health

**Figure 1:**
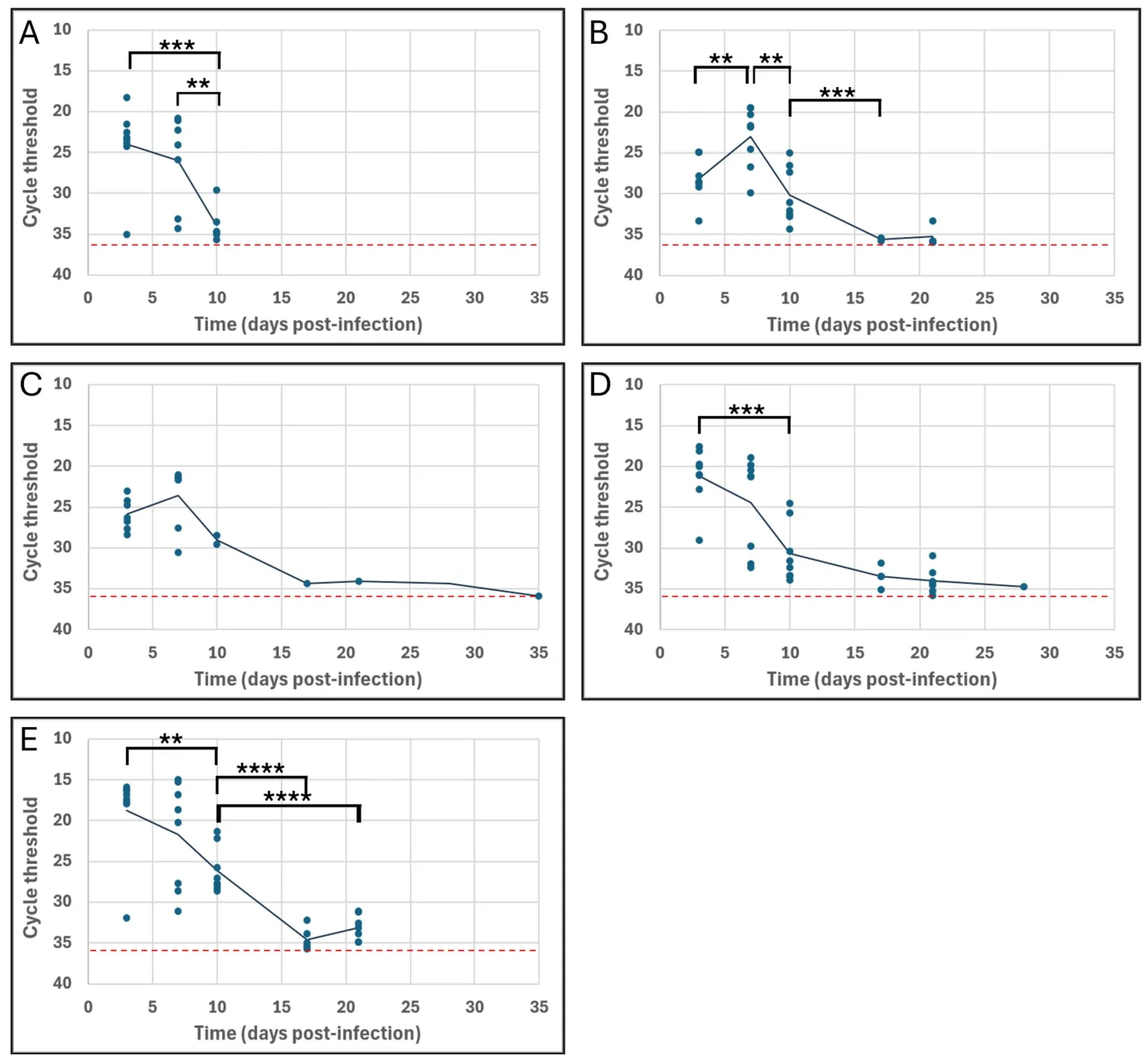
Detection of viral RNA in male mice infected with Ebola virus (EBOV) Makona using RT-PCR with primers for EBOV NP. (**A**) Blood; (**B**) liver; (**C**) testis (seminiferous tubules); (**D**) epididymis; (**E**) sperm. Each data point represents one mouse, and samples from eight mice were collected for each time point. Asterisks indicate a significant change in viral load, where **, *** and **** indicate that *p* < 0.01, *p* < 0.001, and *p* < 0.0001, respectively. Significant differences in time points were determined using the one-way ANOVA test, and significant changes in mean cycle threshold between time points were determined using Student’s *t*-test, for which equality of variance was determined using the F-test. The red dashed line indicates the cutoff for the minimum threshold of detection at 36 cycles.

To assess whether EBOV could translocate to the male reproductive tracts of mice, we infected male *Ifnar1*^−/−^ mice and assessed viral RNA in reproductive tissues and additional locations as shown in Table 1. Signs of illness were mild in the infected mice throughout the course of infection, with none reaching the severity threshold for euthanasia. Viral infection was confirmed based on the detection of EBOV RNA in blood samples by RT-PCR with primer pairs specific to EBOV NP. EBOV RNA was detected in blood samples from all mice sacrificed on day 3, 87.5% of mice sacrificed on day 7, and 75% of mice sacrificed on day 10 (Table 1). Significant changes in mean Ct occurred between day 3 and day 10 (*p* = 5.92 × 10^−4^), and between day 7 and day 10 (*p* = 8.71 × 10^−3^) (Figure 1A). EBOV RNA was also detected in liver samples from 87.5% of mice sacrificed on day 3, all mice sacrificed on days 7 and 10, 37.5% of mice sacrificed on day 17, and 50% of mice sacrificed on day 21 (Table 1). Significant changes in mean Ct occurred between day 3 and day 7 (*p* = 9.50 × 10^−3^), day 7 and day 10 (*p* = 1.27 × 10^−3^), and day 10 and day 17 (*p* = 2.88 × 10^−3^) (Figure 1B).

### 3.2. Ebola Virus RNA Persistence in the Male Reproductive Tract

Viral RNA was detected in testis samples from 87.5% of mice sacrificed on days 3 and 7, 25% of mice sacrificed on day 10, and 12.5% of mice sacrificed on days 17, 21, and 35 (Table 1). Changes in mean Ct between time points for testis samples did not reach thresholds of significance (Figure 1C). Viral RNA was detected in epididymis samples of all mice sacrificed on days 3, 7, and 10, 50% of mice sacrificed on day 17, 87.5% of mice sacrificed on day 21, and 12.5% of mice sacrificed on day 28 (Table 1). Significant changes in mean Ct occurred between day 3 and day 10 (*p* = 1.21 × 10^−4^) (Figure 1D). Viral RNA was detected in sperm samples of all mice sacrificed on days 3, 7, and day 10, in 75% of mice sacrificed on day 17, and in 87.5% of mice sacrificed on day 21 (Table 1). Significant changes in mean Ct occurred between day 3 and day 10 (*p* = 4.22 × 10^−3^), day 10 and day 17 (*p* = 1.93 × 10^−5^) and day 10 and day 21 (*p* = 6.27 × 10^−5^) (Figure 1E).

### 3.3. Histopathology

#### 3.3.1. Liver Tissues

**Figure 2:**
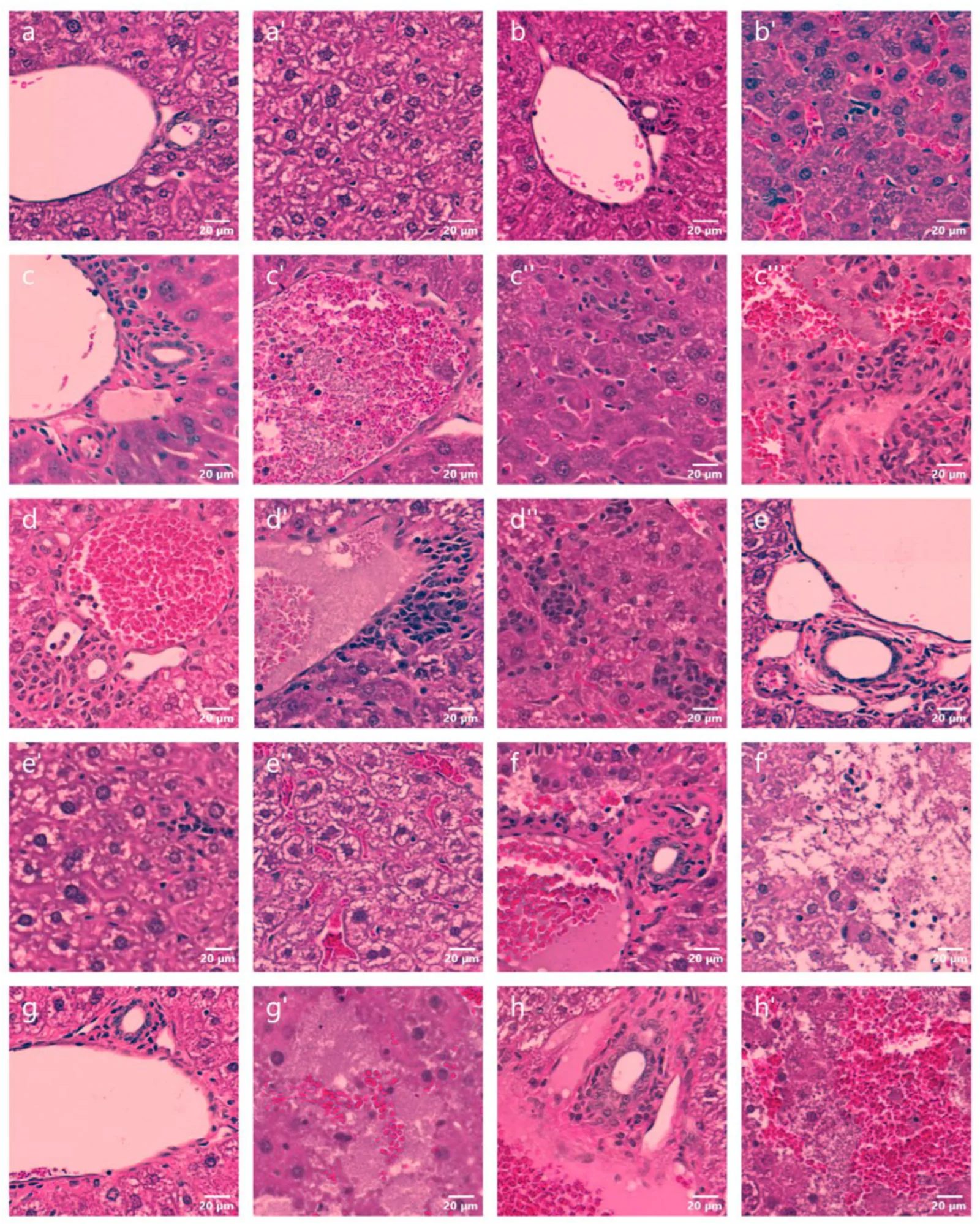
Liver histopathology in male *Ifnar1*^−/−^ mice infected with EBOV Makona. The time point that an image represents is indicated by a lower-case letter, and additional images from the same time point are appended with ′, ″ or ‴: (**a**) negative control mice; (**b**) day 3; (**c**) day 7; (**d**) day 10; (**e**) day 17; (**f**) day 21; (**g**) day 28; (**h**) day 35. Tissues were stained with hematoxylin and eosin, and images are representative of tissue samples from three mice per time point.

Liver tissue sections collected from negative control animals 14 days post-infection showed no signs of infection around veins (Figure 2a) or amongst hepatocytes (Figure 2a′). On day 3 post-infection, increased leukocyte density was observed around bile ducts of portal veins in two mice, but the veins themselves remained relatively free of signs of illness (Figure 2b). Amongst hepatocytes, mild sinusoid congestion was rarely or occasionally observed in two of three mice (Figure 2b′). On day 7 greater histopathology was observed in all mice compared to day 3, although severity varied by mouse. Leukocyte density increased around bile ducts and in proximity to veins (Figure 2c), and veins occasionally or commonly displayed eosinophilic congestion, and to a lesser extent basophilic cells and cell debris (Figure 2c′). Amongst hepatocytes, foci of inflammation were observed more often and with greater severity in all mice compared to day 3, but sinusoid congestion was less severe (Figure 2c″). In one mouse, multiple discrete regions consisting of eosinophilic congestion, increased leukocyte density, and cell debris were observed (Figure 2c‴). On day 10 overall histopathology was greatest compared to other time points; veins of liver tissues from all mice commonly displayed eosinophilic congestion (Figure 2d) and cell debris (Figure 2d′), often in conjunction with more severe proximal inflammation than was observed on day 7. Foci of inflammation amongst hepatocytes were also more common and severe than on day 7 (Figure 2d″). On day 17, overall histopathology was reduced compared to day 10; mice displayed a similar severity of congestion in blood vessels and proximal inflammation, although leukocyte density remained elevated around bile ducts (Figure 2e). No inflammation was observed amongst hepatocytes of two tissue samples, and in the third sample foci were rare and mild (Figure 2e′). Occasional sinusoid congestion was also observed in one sample (Figure 2e″). On day 21, congested veins with proximal hepatocyte apoptosis were noted in one sample (Figure 2f), and cell apoptosis was more widespread in another sample (Figure 2f′). On day 28, increased leukocyte density remained around bile ducts in one sample (Figure 2g), and discrete regions of apoptosis were observed occasionally in one sample and rarely in another (Figure 2g′). On day 35, congested veins and leukocytes proximal to bile ducts were observed in two samples (Figure 2h), one of which also contained regions of hepatocyte apoptosis (Figure 2h′).

#### 3.3.2. Testis Tissues

**Figure 3:**
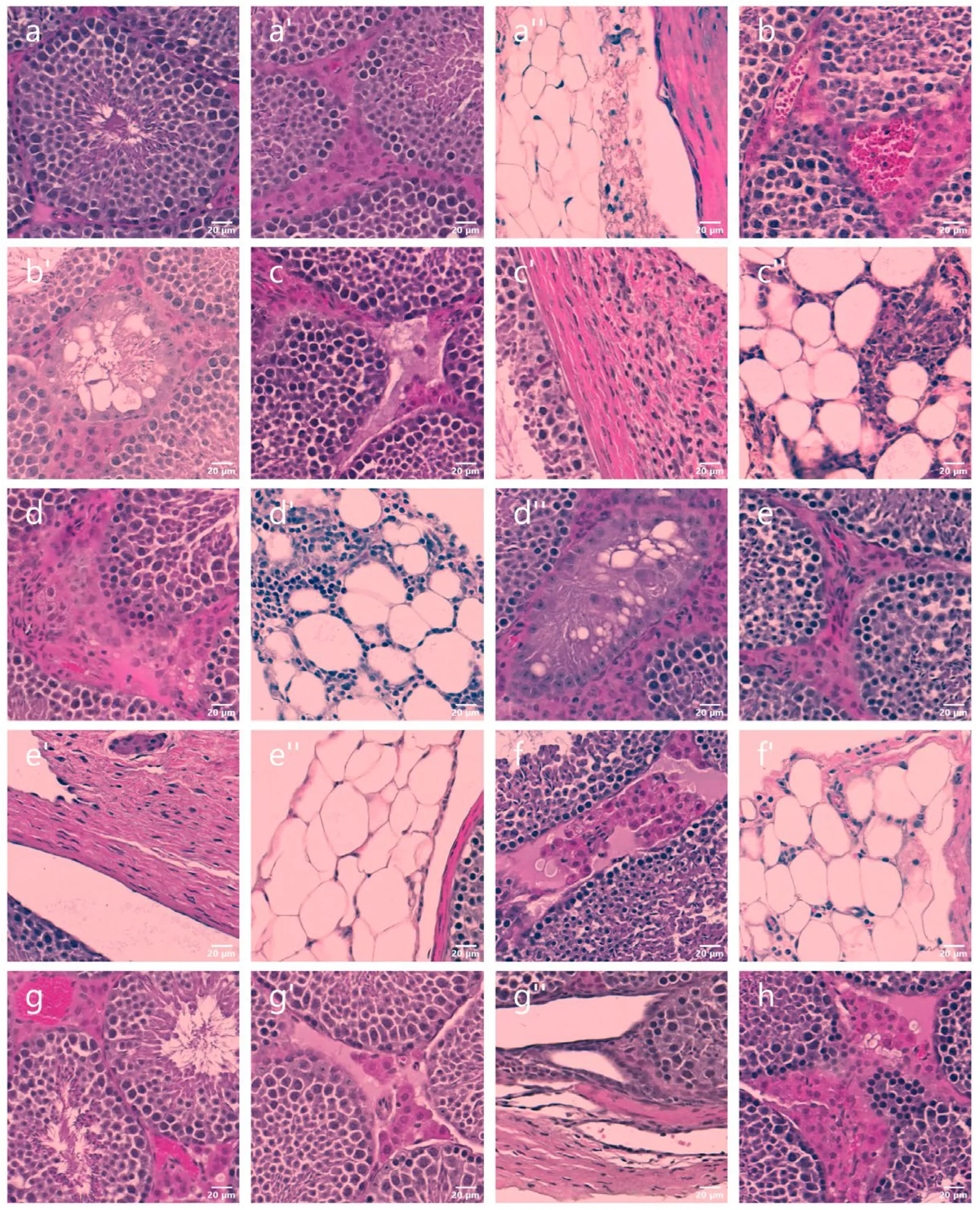
Testis histopathology in *Ifnar1*^−/−^ mice infected with EBOV Makona. The time point that an image represents is indicated by a lower-case letter, and additional images from the same time point are appended with ′, ″ or ‴: (**a**) negative control mice; (**b**) day 3; (**c**) day 7; (**d**) day 10; (**e**) day 17; (**f**) day 21; (**g**) day 28; (**h**) day 35. Tissues were stained with hematoxylin and eosin, and images are representative of tissue samples from three mice per time point.

Testis tissue sections collected from negative control animals 14 days post-infection showed no signs of infection in the seminiferous tubules (Figure 3a), interstitial space (Figure 3a′), or adipose and connective tissue (Figure 3a″). On day 3 post-infection, testis tissues were generally healthy, although interstitial blood vessels were more congested in two samples compared to controls (Figure 3b). One sample was also noted to contain a greater number of seminiferous tubules that displayed signs of necrosis and/or disrupted spermatogenesis (Figure 3b′). On day 7, more vacuolation and cell debris was observed in the interstitial spaces in all samples compared to day 3 (Figure 3c). In addition, elevated leukocyte density and/or foci of inflammation were noted in the tunica albuginea, particularly in areas proximal to the mediastinum (Figure 3c′). Inflammation was observed in adipose tissue and proximal connective tissue in one of three samples (Figure 3c″). On day 10, foci of inflammation in the interstitial space were more common and severe in two samples compared to day 7, but the degree of vacuolation was similar (Figure 3d). Leukocyte density in adipose and connective tissues of all samples was similar or greater on day 10 compared to day 7 (Figure 3d′). Lastly, one sample contained more necrotic seminiferous tubules than expected (Figure 3d″). On day 17, foci of inflammation were rare in all samples (Figure 3e), and leukocyte densities in the tunica albuginea were reduced compared to day 10 (Figure 3e′). Leukocyte densities were also reduced in adipose and connective tissues on day 17 compared to day 10 (Figure 3e″). On day 21, vacuolation and cell debris in the interstitial space was more common than on day 17 (Figure 3f), as was the density of leukocytes in adipose tissues (Figure 3f′). On day 28, blood vessels were commonly congested (Figure 3g), and the frequency of vacuolation and cell debris in the interstitial space was similar to day 21 (Figure 3g′). However, no signs of illness were observed in the tunica albuginea (Figure 3g″). On day 35, vacuolation and cell debris in the interstitial space remained apparent in all samples, although frequency was reduced compared to day 28 (Figure 3h).

## 4. Discussion

In the current model for EVD pathogenesis, macrophages and dendritic cells in blood vessels and lymph nodes are the initial targets of EBOV, followed by the visceral organs and immune-privileged tissues [31]. However, mice infected with wild-type or mouse-adapted EBOV have been reported to harbor viral RNA in visceral organs 1 day post-infection [17]. We used an *Ifnar1*^−/−^ mouse model of EVD to elucidate the timing of EBOV dissemination into the male reproductive tract. We found that relative amounts of viral RNA in testis, epididymis, and sperm samples had peaked by day 3 post-infection, although viral loads in sperm samples and epididymis samples were higher than in testis samples at this point. In comparison, the amount of viral RNA in liver samples was lower on day 3 and did not peak until day 7. Since the amount of viral RNA in blood samples had also peaked by day 3 post-infection, dissemination of EBOV into the male reproductive tract tissues may occur roughly in parallel with the onset of viremia, rather than sometime after infection of visceral organs.

Immune cells are a normal presence in the epididymis but not in seminiferous tubules, so we expected EBOV RNA to be present in epididymis tissue samples earlier as a result of viral infiltration via infected dendritic cells and macrophages [10,11,31,32]. Lower average viral loads in epididymis samples on day 3 compared to testis samples may be indicative of earlier dissemination into the epididymis, and productive viral replication may be aided by the presence of mononuclear phagocytes, but data from samples collected earlier than three days post-infection are necessary for confirmation. However, the average viral loads in testis samples on day 3 were still higher than expected considering that the blood testis barrier is restrictive to immune factors [33]. This may be partially explained by the homogenized nature of our testis samples; EBOV RNA may be present in the blood vessels, adipose and connective tissues prior to less productive infection of seminiferous tubules [34]. Greater resistance of seminiferous tubules to EBOV infection would explain the rapid decline in the proportion of samples that tested positive for EBOV RNA between day 7 and day 10, a point at which all liver and epididymis samples tested positive for EBOV RNA. In either case, the addition of earlier time points in the experiment would provide greater clarity.

Given that testis and epididymis tissues contained high viral loads on day 3 relative to other sample types and time points, it is not surprising that sperm samples contained high viral loads as well. Sperm samples contained higher viral loads than testis samples or epididymis samples until day 10, after which viral loads in sperm samples were similar to those of epididymis samples. As such, sperm cells may be infected as they mature in the epididymis. To a lesser extent, precursor germ cells located in the basal region of the seminiferous tubules may also become infected from EBOV in the interstitial space, after which the infected germ cells could be transported across the blood testis barrier during spermatogenesis [35]. In either case, our findings indicate that EBOV disseminates through the male reproductive tract earlier than may be expected based on the current model for EVD pathogenesis [31]. Future investigation would be necessary to support this proposal.

As part of the current project, we tested EBOV persistence in the reproductive tissues of infected male *Ifnar1*^−/−^ mice. Previous studies utilizing mouse models of EVD describe persistence of EBOV in testis and/or epididymis samples 14–35 days post-infection, and persistent infection of seminiferous tubules and germ cells has been reported for EBOV-infected Rhesus macaques [13,14,26]. Since EBOV RNA was consistently detected in blood samples up to day 10, we considered this to be the end of acute infection and defined EBOV persistence as the presence of viral RNA in samples from day 17 onward. We detected EBOV RNA in reproductive tissues up to day 35, although the frequency of detection in all sample types decreased sharply after day 21. The fact that persistent EBOV infection was not maintained in more mice, or for a longer period of time, may be attributed to less severe illness in our test mice. Based on previous studies, we expected a portion of *Ifnar1*^−/−^ mice challenged with EBOV to succumb to illness [23]. Since no infections were fatal, the severity of EVD in our model was likely lower than intended, which could impact the establishment of persistence [27]. Our use of *Ifnar1*^−/−^ mice may have also affected the frequency and extent of persistence, since the establishment of persistent infections requires a complex balance of factors related to viral transcription and replication kinetics and host cell immune response [34,36,37]. It is also possible that EBOV persists in concentrations below our limit of detection, which may be subject to surges in viral replication that would in turn explain reports of intermittent shedding of EBOV genomic material in semen [6,38,39]. In this case, as-yet-undetermined external stimuli may trigger a return to rapid increases in viral replication and shedding of viable virion particles in semen. As an example, Strong et al. (2008) demonstrated that persistently infected mouse and bat cells could be stimulated to increase infectious viral titre in response to exogenous phorbol-12-myristate-13-acetate or lipopolysaccharide [37]. The authors reason that persistent EBOV maintains a balance with interferon antiviral signaling, and that exogenous stimulation of the Ras/MAPK pathway suppresses interferon signaling to facilitate increases in infectious EBOV production [37].

Apart from the risk that virion particles shed in semen pose as a vector of transmission, EBOV persistence in the reproductive tract may negatively impact the sexual health of the infected individual. In a previous report of mice challenged with mouse-adapted EBOV, authors noted minimal to moderate spermatogonia degeneration in the testis 14 days and 35 days post-infection, respectively [14]. In monkeys with delayed time of death, authors noted degeneration of spermatogonia in the epididymis [13]. Although image quality limited the conclusions that could be drawn about the overall state of spermatogenesis in the seminiferous tubules, disruption of cells in the interstitial space was apparent in tissue samples collected up to day 35. This is notable because germ cell degeneration can be caused by a loss of support cells (e.g., epithelial cell necrosis) and external stimuli such as changes in signaling factors, among others [40]. Since interstitial cells such as Leydig cells play a major role in signaling related to spermatogenesis, persistent damage to these factors could impact the overall sexual health of EVD survivors [41]. Unfortunately, we lacked the sample volumes necessary to determine sperm count, morphology, and motility, and sperm parameter tests have not been applied in studies of human EVD survivors. As such, the extent to which acute and persistent EBOV impacts fertility remains unclear.

In conclusion, we determined that EBOV disseminated through the male reproductive tract and into seminal components roughly concurrently with viremia. In addition, exposure of sperm to EBOV in the earliest stages of the reproductive tract during acute infection is more likely to be the result of productive viral replication in the epididymis rather than the testis. Similarly, the presence of EBOV RNA in sperm samples and the epididymis after acute EVD coincide. Lastly, we demonstrated changes in histopathology in the liver and testis during and after acute EVD, which highlights potential lasting damage to tissues associated with spermatogenesis.

## Figures and Tables

**Table 1:** Number of samples per time point containing EBOV RNA.

Sample Type	Naïve	Day 3	Day 7	Day 10	Day 17	Day 21	Day 28	Day 35
Blood	0	8	7	6	0	0	0	0
Liver	0	7	8	8	3	4	0	0
Testis	0	7	7	2	1	1	0	1
Epididymis	0	8	8	8	4	7	1	0
Sperm	0	8	8	8	6	7	0	0

Ct value cutoff < 36; negative control mice time point *n* = 3; EBOV challenge mice time points *n* = 8.

## Data Availability

Data are available upon reasonable request to the corresponding author.
